# Understanding the Triple Burden of HIV, Noncommunicable Diseases, and Mental Health Conditions Among Female Sex Workers With HIV in the Dominican Republic

**DOI:** 10.1177/23259582261455718

**Published:** 2026-06-03

**Authors:** Clare Barrington, Deshira D. Wallace, Carolina Ruiz, Yeycy Donastorg, Martha Perez, Hoisex Gomez, Yicenia Brito Beltre, Meredith Dockery, Deanna Kerrigan

**Affiliations:** 1 Department of Health Behavior, Gillings School of Global Public Health, 446733 University of North Carolina at Chapel Hill, Chapel Hill, NC, USA; 2 HIV Vaccine and Research Unit, Instituto Dermatológico y Cirugía de Piel Dr Huberto Bogaert Díaz, Santo Domingo, Dominican Republic; 3 Department of Internal Medicine, 303830 Hospital Salvador Gotier, Santo Domingo, Dominican Republic; 4 Department of Health, Behavior, Society & Policy, School of Public Health, 51893 Rutgers University, Piscataway, NJ, USA

**Keywords:** HIV/AIDS, mental health, integrated care, vulnerable populations, country profiles, treatment adherence, outcome measures, women

## Abstract

**Objectives:**

The purpose of this study was to determine the prevalence of type 2 diabetes, hypertension, and mental health conditions among female sex workers with HIV in Santo Domingo, Dominican Republic.

**Methods:**

We conducted a cross-sectional survey, blood draw, and medical exam with women (n = 200) recruited by peer navigators. We determined prevalence and used multivariable logistic regression to assess the relationship between conditions.

**Results:**

Mean participant age was 46.1 years (SD: 9.15). Most participants (89.0%) were virally suppressed. Diabetes prevalence was 7.0% and hypertension was 63.0%. Nearly half reported moderate-to-severe depressive symptoms (46.0%) and over half (65.8%) anxiety. Women with moderate-to-severe depressive symptoms were over 4 times more likely to be virally unsuppressed (adjusted odds ratio [aOR]=4.17 [95% CI: 1.37-12.68]) and over 3 times more likely to have diabetes (aOR=3.45 [95% CI: 1.00-11.91]).

**Conclusion:**

Findings indicate the need for holistic, integrated healthcare models to address multiple health conditions and support overall wellbeing.

## Plain Language Summary

Understanding the Burden of HIV, Noncommunicable Diseases, and Mental Health Conditions Among Female Sex Workers with HIV in the Dominican Republic

People with HIV are aging and increasingly developing noncommunicable chronic diseases (NCDs), which are leading causes of death in Latin America and the Caribbean. We conducted a study with female sex workers with HIV in Santo Domingo, Dominican Republic to determine the prevalence of type 2 diabetes, hypertension, and mental health conditions. We used statistical analysis to describe the levels of each condition and determine the relationship between mental health conditions, viral suppression, and NCDs. Mean participant age was 46.1 years (SD: 9.15). Nearly all participants (89.0%) were virally suppressed. Diabetes prevalence was 7.0% and hypertension was 63.0%. Nearly half reported moderate-to-severe depressive symptoms (46.0%) and over half (65.8%) anxiety. Women with moderate-to-severe depressive symptoms were over 4 times more likely to be virally unsuppressed (adjusted odds ratio [aOR]=4.17 [95% CI: 1.37-12.68]) and over 3 times more likely to have diabetes (aOR=3.45 [95% CI: 1.00-11.91]). Findings indicate the need for holistic, integrated healthcare models to address multimorbidity and support overall wellbeing. Future research is needed to develop and test such models.

## Introduction

The evolution of the health profile of countries in the Latin America and Caribbean (LAC) region has been described as following the theory of epidemiologic transition, which argues that a high burden of mortality and morbidity attributable to acute, infectious disease is gradually replaced by a high burden of mortality and morbidity attributable to noncommunicable diseases (NCDs).^1^ Rather than a process of replacement, however, most LAC countries have a dual burden of infectious diseases, including chronic ones like HIV, and NCDs, including type 2 diabetes (diabetes, hereafter) and hypertension, which are the leading causes of adult death in the region.^2–4^ To date, there has been limited research on the dual burden of HIV and NCDs specifically in the LAC region.

Due to early diagnosis and improvements in the availability and effectiveness of antiretroviral therapy (ART), people with HIV (PWH) are living into older adulthood and increasingly developing age-related NCDs. Beyond age, chronic inflammation caused by HIV and metabolic effects of ART can raise susceptibility to and severity of diabetes and hypertension among PWH. As a result, studies from North America, Asia, and sub-Saharan Africa consistently document a high burden of noncommunicable diseases among PWH.^5–10^ Notably, we identified only one study that included female sex workers with HIV, which similarly documented a high burden of NCDs.^11^ While engagement in HIV care can increase opportunities for NCD screening, such practices frequently lack standardization, and HIV providers may have limited capacity and resources to provide holistic care.^5,6,12^ Weak referral processes within siloed healthcare systems, stigma and provider bias, economic factors, and lack of knowledge and self-efficacy related to self-management are barriers to care for NCDs among PWH.^12–14^ As a result, research across settings has documented better levels of HIV viral suppression than glycemic and blood pressure control among PWH.^15–17^

While mental health conditions were not explicitly considered in the original theory of epidemiologic transition, they are now recognized as a critical part of holistic health and wellbeing^18^ and a major economic burden on health systems in LAC^19^ and specifically among female sex workers.^20^ Results of a systematic review and meta-analysis of mental health among female sex workers in low- and middle-income countries indicated a nearly 42% prevalence of depression and 21% anxiety.^21^ Stressors related to living with and managing chronic conditions include fear of mortality and health complications, barriers in access to care and care-related costs, economic productivity, stigma and shame, lifestyle changes, and medication adherence.^17,22,23^ These stressors can contribute to depression and anxiety, which can negatively affect HIV and NCD self-management, clinical outcomes, and overall wellbeing, thus creating a cyclical dynamic of distress among people with multiple chronic conditions.^17^ Inflammatory pathways have also been proposed as a link between chronic physical conditions, including NCDs and HIV, and mental health conditions, including depression and anxiety,^24–26^ as well as genetic pathways from depression to certain subtypes of cardiovascular disease.^26^

The HIV epidemic in the Dominican Republic (DR) is characterized as concentrated, with adult population-level prevalence <1% but disproportionate levels of HIV among specific subgroups of the population, including cisgender female sex workers, who have an estimated HIV prevalence of 4.6%.^27,28^ The Dominican healthcare system is siloed by disease type. A government-sponsored HIV program provides care and ART through a network of decentralized HIV clinics in both governmental and nongovernmental settings. Documented determinants of poor HIV treatment outcomes among cisgender female sex workers with HIV in the DR include depression, substance use, ART interruption, and various forms and types of stigma related to HIV and sex work.^29–34^ To date, there has been minimal research conducted to assess non-HIV health conditions among female sex workers, who have been the focus of HIV research and surveillance but not research on other chronic conditions.

Reflecting epidemiological patterns across the LAC region, nearly three-quarters (72%) of all deaths in the DR are attributed to NCDs, a 40% increase since 2010.^35^ Recent estimates of diabetes prevalence in the DR range from 8.6% to 13.5%.^36,37^ Nearly half (49%) of Dominican adults are estimated to have hypertension, with only about one-quarter of diagnosed adults having their hypertension under control.^38,39^ Reflecting the dual burden of HIV and NCD, in a 2016 study of PWH initiating ART in Santo Domingo (n = 153), 6% had diabetes and 13% had increased cardiovascular risk defined as ≥10% 10-year Framingham risk score.^40^ There is no parallel system of government-sponsored healthcare specially for NCDs, as there is for HIV, though there have been recent efforts in the primary care sector to improve access to NCD care and treatment.

Understanding the triple burden of HIV, NCDs, and mental health conditions in the LAC context is critical for informing healthcare system reforms, health services quality improvements, and community-level support and capacity building efforts. Syndemic theory is a useful framework to guide such work as it recognizes and addresses the interactions between multiple health conditions as well as the contexts in which they occur.^41^ For example, in a scoping review of the syndemic of HIV and NCDs, Karbasi et al (2025) concluded that stigma, poverty, siloed healthcare systems, and poor quality of care for NCDs reduced overall wellbeing for PWH.^20,42,43^ Women are disproportionately impacted by the syndemic of HIV, NCDs, and mental health conditions, in particular depression but there is not specific research on women sex workers.^42,43^

The purpose of the current study was to characterize the burden of NCDs and mental health conditions among female sex workers with HIV in the DR. We first describe the prevalence of NCDs (diabetes and hypertension), mental health conditions (depression and anxiety), and combinations of NCDs and mental health conditions. We then assess the relationship between mental health conditions and HIV viral suppression, diabetes, and hypertension to improve understanding. Informed by Syndemic theory and our prior work in this setting,^29,44^ we hypothesized that we would find a significant association between mental health conditions and all 3 outcomes.

## Methods

### Study Design and Setting

We conducted a cross-sectional survey, medical screening, and blood draw with 200 women with HIV in Santo Domingo, the capital city of the DR, from October 2024 to April 2025. This study was approved by the Institutional Review Boards of the University of North Carolina in the United States (#23-1187) and the Instituto Dermatológico y Cirugía de Piel Dr Huberto Bogaert Díaz (IDCP) (no number), and the National Council for Bioethics in Health (CONABIOS) (#057-2023) in the DR. All participants provided written informed consent prior to enrolling and were compensated based on local standards upon completing their study visit.

### Recruitment

Recruitment was led by peer navigators who re-engaged participants from a prior cohort study^34^ and identified new participants through community and clinic referrals. Eligibility criteria included: (1) being a cisgender women; (2) at least 18 years of age; (3) confirmed HIV-positive diagnosis; and (4) reported engagement in sex work in the last month (or, for participants who had participated in prior research, reported engagement in sex work in the last month when enrolling in the prior study). Peer navigators screened women for these criteria and referred interested individuals to the study site. There were no exclusion criteria. Following confirmation of eligibility at the study site, a trained interviewer obtained written informed consent in Spanish in a private office at IDPC. In the final sample, 44 participants (22%) were re-engaged and 156 (78%) were newly engaged.

### Data Collection

Interviewer-administered surveys were conducted in Spanish by trained interviewers in private offices at IDCP. Responses were entered into a tablet and stored using REDCap, a web-based survey tool. One 10 mL tube of blood was drawn from each participant by trained IDCP staff for the blood-based assessments included in this study (viral load and glycated hemoglobin [HbA1c]). HbA1c testing was conducted at the IDCP lab. Viral load testing was performed at the Centro Sanitario laboratory using the Roche Amplicor HIV-1 Monitor Test, a polymerase chain reaction (PCR) assay. Study team physicians completed the medical screening, including obtaining blood pressure measurements.

### Measures

#### Outcome Variables

*Viral Suppression:* Participants were determined to be suppressed if they had ≤400 copies/mL and unsuppressed if they had >400 copies/mL

*Type 2 Diabetes*. Diabetes was assessed using HbA1c, which measures average blood glucose levels over the last 2 to 3 months. Diabetes diagnosis was coded based on American Diabetes Association guidelines of HbA1c ≥ 6.5%, prediabetes 5.7% to 6.4%, and normal <5.7.^45^ Diabetes was coded as ≥6.5% or a previous diagnosis and self-reported treatment and no diabetes as <6.4% with no self-reported prior diagnosis.

*Hypertension*. Following the office blood pressure protocol,^46^ blood pressure measurements were collected by a trained clinician 3 times using both arms with 5min rests between each measurement. The mean value of the measurements was calculated and dichotomized according to the American Heart Association designation.^47,48^ Hypertension was coded as having a systolic blood pressure ≥130 mm Hg or diastolic blood pressure ≥80 mm Hg or a previous diagnosis and self-reported treatment and no hypertension as <130 mm Hg systolic or <80 mm Hg diastolic with no self-reported prior diagnosis.

#### Independent Variables

*Depressive Symptoms*. We used a modified version of the 9-item Patient Health Questionnaire (PHQ-9) to assess depressive symptoms.^49^ The PHQ-9 measures depressive symptoms over the past 2 weeks and is scored from 0 (none of the days) to 3 (almost every day). To improve comprehension, we divided item 8 of the PHQ-9, “Moving or speaking so slowly that other people could have noticed? Or the opposite—being so fidgety or restless that you have been moving around a lot more than usual” into 2 items. We retained the higher score between the 2 items to calculate the PHQ-9 score. PHQ-9 scores were calculated by summing responses to all 9 items, with a possible range of 0 to 27. Cronbach's alpha was 0.81. Depressive symptoms were coded as moderate-to-severe depressive symptoms (≥10) and none-to-mild depressive symptoms (<10).

*Anxiety Symptoms.* Anxiety symptoms in the past 2 weeks were measured using the anxiety subscale of the Hospital Anxiety and Depression Scale (HADS-A).^50^ The HADS-A includes 7 items that measure anxiety symptoms scored from 0 (none of the days) to 3 (almost every day). HADS-A scores were calculated by summing responses to all 7 items, with a possible range of 0 to 21. Cronbach's alpha was 0.81. Anxiety symptoms were coded as no anxiety or borderline (0-10) and anxiety (≥11).

#### Control Variables

*Time Since HIV Diagnosis.* Participants self-reported the year of their HIV diagnosis. Time since HIV diagnosis was analyzed as a continuous variable.

*Sociodemographic and behavioral factors.* Self-reported sociodemographic variables included age in years, highest level of educational attainment, mean income over the past 6 months, and partner status (eg, single, living with partner). Lifetime history of substance use (yes/no) included marijuana, crack, cocaine, heroin by injection, and pills.

### Statistical Analysis

We first calculated descriptive statistics for all variables. Categorical variables were summarized as frequencies and percentages, and continuous variables as means with standard deviations (Table 1). Our study was sufficiently powered to reject the null hypothesis that the prevalence in our study cohort is the same as the national adult prevalence estimates in DR. Detection levels were set at 17.8% for diabetes and 45.1% for hypertension.

**Table 1.** Characteristics of the Study Population (n = 200).

Characteristics	N(%) or Mean(SD)
Age (years)	46.1(9.15)
Relationship status	
Single	148 (74.0)
Living with partner	52 (26.0)
Average monthly income over past 6 months (DOP)	9844.53^‡^ (13,275.56)
Highest level of education completed	
Never attended school	21 (10.5)
Primary school	76 (38.0)
Secondary school	92 (46.0)
University	11 (5.5)
Depressive symptoms, PHQ-9	
None-mild (<10)	108 (54.0)
Moderate-severe (≥10)	92 (46.0)
Anxiety symptoms, HADS-A*	
None-borderline (≤10)	68 (34.2)
Anxiety (≥11)	131 (65.8)
Substance use	
Ever	16 (8.0)
Never	184 (92.0)
Average time since HIV diagnosis (years)	14.5 (7.4)
ART use	
Never on ART	2 (1.0)
Not currently on ART	5 (2.5)
Currently on ART	193 (96.5)
ART adherence in previous 4 days^†^	
No missed doses	150 (77.7)
Missed doses	43 (22.3)
ART interruption, previous 6 months^†^	
Yes	22 (11.4)
No	171 (88.6)
HIV viral load	
Unsuppressed (≥400 copies/mL)	22 (11.0)
Suppressed (<400 copies/mL)	178 (89.0)
Blood glucose levels (HbA1c, %)	
Normal (<5.7%)	133 (66.5)
Prediabetes (5.7%-6.4%)	53 (26.5)
Diabetes (6.5%)	14 (7.0)
Blood pressure category	
Normal	74 (37.0)
Hypertension (≥130/80 mm Hg)	126 (63.0)
Patterns of health conditions	
Diabetes + hypertension	14 (7.0)
Diabetes + depression	9 (4.5)
Diabetes + anxiety*	8 (4.0)
Hypertension + depression	55 (27.5)
Hypertension + anxiety*	82 (41.0)
Diabetes + hypertension + depression	9 (4.5)
Diabetes + hypertension + anxiety*	8 (4.0)
Diabetes + hypertension + depression + anxiety*	6 (3.0)

*: n = 199.

† : n = 193.

‡ Equivalent to approximately USD $164.

Abbreviations: ART, antiretroviral therapy; DOP, Dominican pesos; HADS-A, Hospital Anxiety and Depression Scale - Anxiety subscale; HbA1c, glycated hemoglobin; PHQ-9, Patient Health Questionnaire.

To identify factors associated with each outcome (viral suppression, HbA1c, and blood pressure), we fit separate multivariable logistic regression models. We first determined unadjusted odds ratios using bivariate logistic regression. We then included sociodemographic and HIV-relevant control variables, including age, time since HIV diagnosis, educational attainment, civil status, income, and substance use. This stepwise modeling approach was chosen to maximize parsimony while controlling for potential confounders identified based on prior research and theory. Adjusted odds ratios (aORs) are reported with 95% confidence intervals (CIs) and 2-sided *P*-values. Missing data accounted for less than 5% of the total sample; thus, we employed a case-wise deletion approach to address missingness. Analyses were performed using Stata/SE 19.0.^51^

We used the STROBE reporting guidelines and reporting checklist when drafting and editing this manuscript (Supplemental A).^52^

## Results

### Participant Characteristics

Mean age was 46.1 years (standard deviation [SD] 9.15) (Table 1). Most participants were single (74.0%) and reported an average monthly income of RD$9844.56 Dominican pesos (SD: RD$13,275.56), equivalent to approximately US$164. Nearly half (46.0%) of the participants had completed up to secondary school.

Almost half of the sample (46.0%) reported moderate-to-severe depressive symptoms and 65.8% of participants reported anxiety symptoms. Participants reported low substance use, with 92.0% indicating no history of any drug use.

Mean time since HIV diagnosis was 14.5 years (SD: 7.4). Nearly all participants (96.5%) were on ART at the time of the study. Among those on ART, 77.7% had not missed a dose in the past 4 days and 88.6% had not experienced ART interruption in the previous 6 months. Most (89.0%) were virally suppressed (<400 copies/mL).

Over half (66.5%) of participants were categorized as having normal HbA1c (<5.7%), 26.5% in the prediabetes range (5.7%-6.4%), and 7.0% were within the diabetes range (≥ 6.5%). Three of the 14 participants with diabetes (21.4%) reported no prior diagnosis or treatment for diabetes. Five of the 14 participants with diabetes (35.7%) had an HbA1c below 6.5% and reported a prior diagnosis and being currently in treatment, which was also confirmed during the medical screening component of the study. The remaining 6 participants (42.8%) reported a prior diabetes diagnosis and being in treatment, with physician confirmation, but still had an elevated HbA1c.

Based on the blood pressure assessment, 63.0% of participants were categorized as having hypertension (≥130/80 mm Hg) and 37.0% as normal. Among those identified with hypertension, 59.5% reported no prior diagnosis or treatment for hypertension. Only 3 participants (2.4%) had a prior diagnosis and had their hypertension under control. Finally, 38.1% had a prior diagnosis but still elevated blood pressure.

### Patterns of Multiple Health Conditions

Over a quarter of participants (27.5%) had hypertension and moderate-to-severe depressive symptoms and 41.0% had hypertension and anxiety symptoms. All participants with diabetes (7.0%) also had hypertension. A small proportion (4.5%) had diabetes and depressive symptoms and diabetes and anxiety was (4.0%). Across 3 or more conditions, the prevalence of diabetes, hypertension, and depressive symptoms was 4.5% and for diabetes, hypertension, and anxiety symptoms was 4.0%. About 3.0% of participants had diabetes, hypertension, depressive and anxiety symptoms.

### Associations Between Mental Health and Viral Suppression

In adjusted models (Table 2), participants who reported moderate-to-severe depressive symptoms were significantly more likely to be virally unsuppressed compared to participants who reported no-to-mild depressive symptoms (aOR=4.17 [1.37-12.68]). Anxiety symptoms were not associated with viral suppression.

**Table 2.** Unadjusted and Adjusted Analyses of Factors Associated With Not Being Virally Suppressed (n = 200).

	Unadjusted Models		Model 1*	Model 2^†^
	OR (95% CI) *P*-value		aOR (95% CI) *P*-value	
Moderate-to-severe depressive symptoms	*3.57* *(1.34, 9.58) P* *=* *.01*		*3.20* *(1.18, 8.66) P* *=* *.02*	*4.17* *(1.37, 12.68) P* *=* *.01*
Anxiety symptoms	– 2.55 (0.83, 7.86) *P* = .10		2.09 (0.66,6.60) *P* = .21	1.87 (0.57, 6.13) *P* = .30
Age				0.99 (0.93, 1.05) *p* = 0.68
Average time living with HIV				0.95 (0.88, 1.03) *p* = 0.19
Educational attainment				1.03 (0.38, 2.81) *p* = 0.96
Civil status				0.82 (0.29, 2.81) *p* = 0.72
Income				1.00 (1.00, 1.00) *p* = 0.13
Substance use				0.37 (0.04, 3.16) *p* = 0.37
N	200	199	199	199

*Model 1 is an adjusted model comprised of both moderate-to-severe depressive symptoms and anxiety symptoms.

† Model 2 is an adjusted model comprised of moderate-to-severe depressive symptoms, anxiety symptoms, age, years living with HIV, educational attainment, civil status, income, and substance use.

Abbreviations: aOR, adjusted odds ratio; CI, confidence interval.

### Associations Between Mental Health and Diabetes

The unadjusted models examining associations between moderate-to-severe depressive symptoms and anxiety symptoms and diabetes were not significant (Table 3). However, when adjusting for anxiety symptoms and control variables, participants with moderate-to-severe depressive symptoms were significantly more likely to have diabetes compared to those who reported none-to-mild depressive symptoms (aOR=3.45 [1.01,11.91]). Older participants were also significantly more likely to have diabetes than younger participants (aOR=1.08 [1.00-1.17]).

**Table 3.** Unadjusted and Adjusted Analyses of Factors Associated With Diabetes (n = 200).

	Unadjusted Models		Model 1*	Model 2^†^
	OR (95% CI) *P*-value		aOR (95% CI) *P*-value	
Moderate-to-severe depressive symptoms	2.23 (0.72, 6.92) *P* = .16		2.47 (0.78, 7.85) *P* = .13	*3.45* *(1.00, 11.91) P* *=* *.05*
Anxiety symptoms	– 0.67 (0.22, 2.02) *P* = .48		0.56 (0.18, 1.75) *P* = .32	0.72 (0.21, 2.42) *P* = .59
Age				*1.08* *(1.00, 1.17) P* *=* *.05*
Average time living with HIV				1.02 (0.94, 1.10) *P* = .71
Educational attainment				1.76 (0.54, 5.72) *P* = .35
Civil status				2.94 (0.90, 9.60) *P* = .07
Income				1.00 (1.00, 1.00) *P* = .30
Substance use				1.21 (0.14, 10.86) *p* = 0.86
N	200	199	199	199

*Model 1 is an adjusted model comprised of both moderate-to-severe depressive symptoms and anxiety symptoms.

† Model 2 is an adjusted model comprised of moderate-to-severe depressive symptoms, anxiety symptoms, age, years living with HIV, educational attainment, civil status, income, and substance use.

Abbreviations: aOR, adjusted odds ratio; CI, confidence interval.

### Associations Between Mental Health and Hypertension

Depressive symptoms and anxiety were not associated with hypertension (Table 4). Older participants were significantly more likely to have hypertension than younger participants (aOR=1.08 [1.04-1.13]).

**Table 4.** Unadjusted and Adjusted Analyses of Factors Associated With Hypertension (n = 200).

	Unadjusted Models		Model 1*	Model 2^†^
	OR (95% CI) *P*-value		aOR (95% CI) *P*-value	
Moderate-to-severe depressive symptoms	0.77 (0.44, 1.38) *P* = .39		0.78 (0.44, 1.41) *p* = .41	1.03 (0.54, 1.95) *P* = .94
Anxiety symptoms	– 0.97 (0.53, 1.78) *P* = .93		1.02 (0.55, 1.89) *P* = .95	1.24 (0.63, 2.41) *P* = .53
Age				*1.08* *(1.04, 1.13) P* *=* *0.00*
Average time living with HIV				1.02 (0.97, 1.07) *P* = .44
Educational attainment				1.64 (0.86, 3.15) *P* = .14
Civil status				1.51 (0.78, 2.93) *P* = .22
Income				1.00 (1.00, 1.00) *P* = .38
Substance use				0.97 (0.32, 2.94) *P* = .95
N	200	199	199	199

*Model 1 is an adjusted model comprised of both moderate-to-severe depressive symptoms and anxiety symptoms.

† Model 2 is an adjusted model comprised of moderate-to-severe depressive symptoms, anxiety symptoms, age, years living with HIV, educational attainment, civil status, income, and substance use.

Abbreviations: aOR, adjusted odds ratio; CI, confidence interval.

## Discussion

We assessed the prevalence of noncommunicable diseases (NCDs), specifically diabetes and hypertension, and mental health conditions (depressive and anxiety symptoms) among female sex workers with HIV in Santo Domingo, Dominican Republic. Overall, we found high levels of all assessed NCDs and mental health conditions. While lower than the national estimate of 13.45% diabetes prevalence,^37^ our finding of 7.0% diabetes prevalence was consistent with a recent study in the DR that found 8.6% among adults in agricultural areas.^36^ It was also consistent with the only published study on diabetes among PWH in the DR, which reported 6.0% diabetes prevalence.^40^ One interpretation of the lower level of diabetes in our sample could be that our participants enact self-care practices, such as a balanced diet and regular physical activity, as part of their HIV self-management. However, we found 63.0% hypertension, substantially higher than a recent study with Dominican adults, which reported 30.5%, though our study used a lower cutoff of ≥130/80 mm Hg in accordance with the updated American Heart Association guidelines.^38,53^ Beyond the DR, we identified only one study focused on determining the prevalence of NCDs among a sample of mostly female sex workers in Kenya.^11^ In this sample, 18.3% had any NCD, most of which (91.8%) was hypertension with no cases of diabetes. There is a need for further research to expand understanding of the determinants beyond age of both diabetes and hypertension among female sex workers and other PWH to inform tailored efforts to promote preventive and self-management behaviors.

By triangulating survey, biological, and clinical data, we identified gaps in detection and control for both diabetes and hypertension. Undetected cases of diabetes and hypertension may reflect insufficient screening in the context of routine HIV care, as well as the effects of HIV stigma serving as a barrier to engagement in primary healthcare among PWH.^9^ The effect of HIV stigma could be exacerbated among female sex workers who experience additional forms of stigma, for example related to sex work, which can limit access to care and is associated with poor health outcomes.^34^ The high level of uncontrolled diabetes and hypertension is also consistent with research from the LAC region, specifically in Mexico City, where people with both HIV and diabetes had high viral suppression (94%) but only 43% had an HbA1c < 7.^15^ In recent studies across diverse settings, PWH and other chronic conditions have been found to have more hospitalizations, worse retention in care, and lower health-related quality of life than PWH alone.^9,54^ These findings indicate that context-specific research is needed within health systems across LAC to determine opportunities to reduce stigma and improve screening, referrals, and treatment for NCDs as part of comprehensive, integrated care for PWH.

The finding that nearly 90% of our sample was virally suppressed is very encouraging, along with the high levels of adherence. These findings may reflect the effects of improved access to ART, effective health education, and supportive providers, peers, and social networks in the DR.^34,55,56^ The highest level of viral suppression documented in prior research with female sex workers with HIV in this setting was 76.2%.^34^ One potential implication of this finding is to consider integration of care into existing HIV clinics, which have demonstrated the ability to reach and retain PWH as well as achieve optimal HIV outcomes. Chhoun et al (2017) recommended integrating NCD screening into HIV care in Cambodia, which, like the DR, has a concentrated HIV epidemic affecting specific subgroups of the population who may experience stigma in healthcare settings outside of the HIV system.^10^

We documented a high burden of mental health conditions. In prior research with female sex workers with HIV in this setting, 38.0% of participants had moderate-to-severe depression, which is substantially lower than our findings of 46.0%.^29^ We also found that depressive symptoms were significantly associated with both viral suppression and diabetes, highlighting the importance of mental health for the overall wellbeing of women with HIV. Depressive symptoms have been associated with poor HIV outcomes in the DR and other settings.^20^ Support for depression has been identified as a gap in existing HIV care frameworks.^12^ As PWH continue to age, there is a need to identify sustainable strategies to integrate support for depressive symptoms into HIV care systems. Research in the LAC region has documented how even low-intensity psychological support can have a substantial impact on internalized stigma, self-esteem and overall wellbeing of PWH, which could provide models to consider for adaptation and transfer across settings.^57–59^

Our finding of 65.8% anxiety symptoms was also higher than the 18.1% documented in prior research in this setting.^29^ While not associated with any of the other health outcomes, this is an alarming finding and a call to action to expand research and support for anxiety. In the Dominican setting, post-COVID economic stressors have been anecdotally identified as a potential driver of heightened anxiety, which is an area requiring empirical assessment. Due to the cross-sectional nature of our data, we cannot establish temporality, which is important given the emerging work linking genetic and inflammatory mechanisms to depression and chronic disease.^26^ Additionally, while we asked participants about engagement in care for diabetes and hypertension, we did not ask about participation in mental health support services or treatment, which is critical to understand to inform effective responses.

Finally, our findings that women with HIV also have high levels of NCDs and mental health conditions reflects the aforementioned Syndemic theory, which refers to the interactions between multiple health conditions and the conditions in which they occur.^41^ Our findings echo recent studies reporting higher levels of NCDs among PWH *and* mental health conditions than HIV alone.^60,61^ Studies have also reported a higher burden of stress and depression among PWH and cardiovascular conditions, compared to HIV alone, highlighting the negative implications of the triple burden for mental health and wellbeing.^17,22^ Salimu et al (2025) found that limited health literacy, low self-efficacy, and inadequate structural support transcended experiences with both HIV and NCDs, which provides some insight into the conditions surrounding the syndemic of HIV, NCDs, and mental health.^17^ Beyond describing patterns of disease, there is a need for context-specific research to explore the dynamics surrounding vulnerability and the opportunities within health systems to provide more comprehensive care, especially among populations such as female sex workers who experience multiple, intersecting forms of stigma.^34^

This study had several strengths and limitations. Strengths included use of survey and biological data to obtain a comprehensive understanding of the holistic health profiles of female sex workers with HIV. We used robust, reliable measures that have been used in prior research in this setting and with the same population. Strong, longstanding community collaborations informed study procedures and facilitated appropriate engagement. However, the cross-sectional nature of the current study does not allow for the determination of causal relationships and we identified a lower prevalence of diabetes than hypothesized, which may limit power. We re-engaged participants from prior research and recruited new participants using nonrandom approaches, which may have introduced bias into the study. We assessed HbA1c levels to determine diabetes, which in most cases is a robust measure of glycemic control over time. However, there is well-documented evidence that ART can affect the reliability of HbA1c measurements, which may have affected the classification of diabetes status in our sample.^62^ Future research could use fasting glucose plasma tests to assess blood glucose levels, though this often requires an early morning appointment, which could be a challenge for our study population.

## Conclusions

Female sex workers living with HIV in the DR are simultaneously managing multiple competing health demands, including NCDs and mental health conditions. There is an urgent need for an integrated care model that addresses these holistic health needs to promote optimal health and wellbeing. Findings highlight the success of the existing HIV treatment model, which could serve as a platform for an integrated model to provide holistic care for HIV, NCDs, and mental health conditions and support overall wellbeing and quality of life. Future research is needed to determine the pathways between depressive symptoms and health outcomes.

## Supplemental Material

sj-docx-1-jia-10.1177_23259582261455718 - Supplemental material for Understanding the Triple Burden of HIV, Noncommunicable Diseases, and Mental Health Conditions Among Female Sex Workers With HIV in the Dominican Republic Supplemental material, sj-docx-1-jia-10.1177_23259582261455718 for Understanding the Triple Burden of HIV, Noncommunicable Diseases, and Mental Health Conditions Among Female Sex Workers With HIV in the Dominican Republic by Clare Barrington, Deshira D. Wallace, Carolina Ruiz, Yeycy Donastorg, Martha Perez, Hoisex Gomez, Yicenia Brito Beltre, Meredith Dockery and Deanna Kerrigan in Journal of the International Association of Providers of AIDS Care (JIAPAC)

sj-docx-2-jia-10.1177_23259582261455718 - Supplemental material for Understanding the Triple Burden of HIV, Noncommunicable Diseases, and Mental Health Conditions Among Female Sex Workers With HIV in the Dominican Republic Supplemental material, sj-docx-2-jia-10.1177_23259582261455718 for Understanding the Triple Burden of HIV, Noncommunicable Diseases, and Mental Health Conditions Among Female Sex Workers With HIV in the Dominican Republic by Clare Barrington, Deshira D. Wallace, Carolina Ruiz, Yeycy Donastorg, Martha Perez, Hoisex Gomez, Yicenia Brito Beltre, Meredith Dockery and Deanna Kerrigan in Journal of the International Association of Providers of AIDS Care (JIAPAC)
