# Supplementary material for: Understanding the Triple Burden of HIV, Noncommunicable Diseases, and Mental Health Conditions Among Female Sex Workers With HIV in the Dominican Republic
Source: J Int Assoc Provid AIDS Care. 2026 Jun 3;25:23259582261455718. doi: 10.1177/23259582261455718 (PMC13237256; doi:10.1177/23259582261455718)
Supplement: sj-docx-2-jia-10.1177_23259582261455718 - Supplemental material for Understanding the Triple Burden of HIV, Noncommunicable Diseases, and Mental Health Conditions Among Female Sex Workers With HIV in the Dominican Republic [file sj-docx-2-jia-10.1177_23259582261455718.docx]

**Present the study to potential participants:**

*Instructions to interviewer(s):*

Text that appears in italics is not read to the participant.

All other text should be read to the participant (word for word).

Text in [brackets] are probes or additional prompts.

***Introduce the study to potential participants:***

The Institute for Dermatology and Skin Surgery (IDCP) in the Dominican Republic and the University of North Carolina in the United States are collaborating to conduct a project called Understanding the Experience of Living with Diabetes and Hypertension in Sex Worker Women Living with HIV in the Dominican Republic to improve integration of care. As explained to you in the consent, this study is about the experience of HIV and other chronic diseases such as hypertension and diabetes in women with HIV.

I would like to ask you a few questions to determine if you are eligible to participate in this study: Can I continue? Yes/No

**Table of contents of the survey:**

Section I: Administrative

Section II: Demographic and Economic Indicators

Section III: Living with HIV

Section IV: Other Health Conditions

Section V: Mental Health

Section VI: Substance Use

Section VII: Sex Work

Section VIII: Sexual Partnerships

Section IX: Cohesion, Social Support, and Social Participation

Section XI: Exposure to Intervention Activities

**FORM TO DETERMINE PARTICIPANT ELIGIBILITY**

| **Eligibility** | | | | | | |
| --- | --- | --- | --- | --- | --- | --- |
| **Vairable Purpose** | **Variable Name** | | **Question** | **Response Options** | | **Logic** |
| Eligibility | recruit_DR | | *Choose the option on where/how you recruited the participant* | Referred by navigator  Referred by another participant  Referred by SAI  Identified by IDCP staff  Other:_____________ | | Required |
| Eligibility | consent | | ***Interviewer Question:*** *Does the participant appear capable of providing informed consent (i.e., the person does not have a permanent physical disability (i.e., the person is deaf) or a mental disability that would prevent the person from providing consent? [Interviewer indicated Yes or No].* | Yes  No | |  |
|  | Consent3 | | ***Interviewer Question:*** *Did this person participate in the social determinants cohort?* | Yes  No | |  |
| Eligibility | screen_HIV | | *Interviewer Question: Has it been confirmed that the participant lives with HIV by a confirmatory rapid test?* | Yes, by rapid confirmatory test  No | | Required |
| Eligibility | age_elig | | Are you at least 18 years old? | Yes  No | | Required |
| Eligibility | sw_30days | | Have you exchanged sex for money in the last month? | Yes  No | | Required |
| Eligibility | eligible | | ***Interviewer Question:*** *Is this person eligible for the study?*      *If the answer to all of the eligibility questions was YES, the person is eligible.*    *If the answer was NO to at least one of the eligibility questions, the person is not eligible.* | Yes  No | | Required |
| You are **eligible** to participate in our study.  We will now give you information about our study and ask for your consent to participate.  [BEGIN THE INFORMED CONSENT PROCESS]. | | | | | | Show if “eligible” is YES |
| You are **NOT eligible** to participate in our study.  We are looking for people with certain characteristics that are needed at the time of the survey.  If you require additional information, we can provide you with information about health services in your area.  *Thank participant for their time and close the survey session.* | | | | | | Show if “eligible”=NO |
| Eligibility | consent2 | *Did the person give consent to participate in the study?* | | | Sí  No | Show if "eligible"=YES;  if consent2=YES, show message “Proceed with blood test and then survey.”;  If consent2=No, write the reason below |
| *If the answer is* ***NO*** *thank the participant for their time and close the survey session.* | | | | | | Show if consent2=NO |

**Instructions for interviewer:**
Text that appears in ***italics*** is not read to the participant.
All other text should be read to the participant (word for word).

Text in brackets are probes and optional.

## Section I: Administrative

| **Variable Purpose** | **Variable Name** | **Question** | **Response Options** | **Logic** |
| --- | --- | --- | --- | --- |
| Administrative | int_code | *Interviewer Code:* | Initials (3 spaces)  (i.e., MPM, HGM, YDC) | REQUIRED |
| Administrative | int_date | *Date of Interview:* | [dd][mm][yy] |  |
| Administrative | int_start | *Interview start time:* | [HH][MM] |  |
| Administrative | mt_ptid | *Participant identification code VIHDBDB + [3 digits] (e.g. VIHDB001):* | VIHDB+ [3 digits]  (e.g., VIHDB) | REQUIRED |

## Section II: Demographic and Economic Indicators

| **Variable Purpose** | **Variable Name** | **Question** | **Response Options** | **Logic** |
| --- | --- | --- | --- | --- |
| **Demographic Data** | | | | |
| **Statement to read to participant before questions:** | | **To start, I will be asking you some questions about yourself.** | | |
| Demographics | age_1 | What is your date of birth? | [dd][mm][yyyy] |  |
| Demographics | age_2 | How old are you?  If you don't know exactly, please tell me what you have been told or what you think. The program automatically displays the participant's age, check it against the age you gave.    *If the age difference is more than 2 years, ask the participant to confirm.* | _ _ years | Calculate the age based on the year and check against the age given.    If the age difference is >2 years, ask the participant to confirm. |
| Demographics | born | Where were you born? | Santo Domingo  Another city / town in the interior  Haiti  Other (specify): _________ |  |
| Demographics | reside | Currently, you reside in: | Santo Domingo  Another city / town in the interior  Haiti  Other (specify): _________ |  |
| Demographics | change_loc | Have you moved to another city in the last 12 months? | Yes  No |  |
| Demographics | marital | What is your marital status? | Single/never married  Married with papers and living together  Living with someone without papers  Separated  Divorced  Widowed |  |
| Demographics | Live_alone | Do you live alone? | Yes  No |  |
| Demographics | live_sit | Who do you live with at this time?  [Mark all that apply] | Partner (including spouse)  Parents  Children  Other members of the family  Friend(s)  Other (specify)_______ | Show if live_alone is No  Allow for selection of multiple responses |
| Demographics | type_home | At this moment you live in: | Apartment or house you own  Apartment or house you rent  Room rented in a house  Room rented in a pension  Other (specify): ________ |  |
| Demographics | edu1 | Have you ever attended school? | Yes  No |  |
| Demographics | edu2 | Are you currently attending school? | Yes  No | Show if edu1=Yes |
| Demographics | edu3 | What grade are you in? | Primary: 1 2 3 4 5 6 7 8  Secondary: 1 2 3 4  University: 1 2 3 4  Postgraduate school | Show if edu2=Yes |
| Demographics | edu4 | To what grade did you attend? | Yes  No |  |
| Demographics | edu5 | Have you taken any technical courses? | Yes  No |  |
| Demographics | edu6 | Are you currently taking a technical course? | Yes  No | Show if edu5=Yes |
| **Statement to read participants before questions:** | | Now I am going to ask you some questions about the trips you have taken in the last 6 months. | | |
| MIgration | trvel1y4_frq | In the last 6 months, how often did you travel outside of Santo Domingo? | Several times a week  Once a week  Several times a month  Once a month  Less than once a month |  |
| Migration | sw_mig_frq | In the last 6 months, how often did you travel outside of Santo Domingo specifically to exchange sex for money in another place? | Several times a week  Once a week  Several times a month  Once a month  Less than once a month |  |
| **Economic Data** | | | | |
| **Statement to read participant before questions:** | | **Now I’ll ask you some questions about your different forms of income.** | | |
| Economic | percepsec | In the past 6 months, how have you been economically? | Excellent  Very Good  Good  Fair  Poor |  |
| Economic | wrk_lvl | In the past 6 months, which of the following best describes your employment status? | 1 Formal employment  2 Self-employment  3 Not employed/not working |  |
| Economic | occup | In the past 6 months, how do you earn money?  [Mark all that apply] | Sex Work  Waitress  Dancer  Salon or nail center  Other (specify)_______ | Show if wrk_lvl=1 or 2 |
| Economic | id_incom | Normally how much money do you earn/receive per month? | RD$______ |  |
| Economic | incmresp | Are you the main person responsible for supporting your household? | Yes  No |  |
| Economic | spprtoth | How many people other than yourself do you financially support with what you earn/receive? | _______ (number of people) |  |
| Economic | savings1 | Do you normally save some money on a monthly basis? | Yes  No |  |
| Economic | savings2 | How much money do you save on a monthly basis? | RD$______ | Show if savings1=Yes |

## Section III: Living With HIV

| **Variable Purpose** | **Variable Name** | **Question** | **Response Options** | | **Logic** |
| --- | --- | --- | --- | --- | --- |
| **Statement to read participant before questions:** | | **Now I would like to ask you a few questions about your experiences since your first HIV positive test.** | | | |
| **Diagnosis and Disclosure of HIV Status** | | | | | |
| PLHIV | fst_test | In what year were you diagnosed HIV positive (e.g. 1992, 2010)? | _____ YYYY | |  |
| HIV disclosure | hivdisclos3 | Have you told anyone that you are HIV positive (other than your healthcare provider)? | Yes  No | |  |
| HIV disclosure | hivdisclos4 | Who did you tell?  *[Mark all that apply]* | Sexual partner  Sister/female cousin  Brother/male cousin  Mother/aunt  Grandmother/great-aunt  Grandfather/great-uncle  Child  Friend  Other (specify): | | Show if hivdisclos3==”yes” |
| **Internalized HIV-Related Stigma** | | | | | |
| **Statement to read participant before questions:** | | **In this section I’m going to read you some statements about how you may feel about yourself and your HIV status. I would like you to tell me your level of agreement or disagreement with each statement.** | | | |
| PLHIV internalized Stigma scale  *Berger (short) | sf_stig1 | Having HIV makes you feel like a bad person. | 4, Strongly agree  3, Agree  2, Disagree  1, Strongly disagree | |  |
| PLHIV internalized Stigma scale  *Berger (short) | sf_stig2 | You feel like you are not as good as others because you have HIV. | 4, Strongly agree  3, Agree  2, Disagree  1, Strongly disagree | |  |
| PLHIV internalized Stigma scale  *Berger (short) | sf_stig3 | Having HIV makes you feel unclean. | 4, Strongly agree  3, Agree  2, Disagree  1, Strongly disagree | |  |
| PLHIV internalized Stigma scale  *Berger (short) | sf_stig4 | Having HIV in your body is disgusting to you. | 4, Strongly agree  3, Agree  2, Disagree  1, Strongly disagree | |  |
| PLHIV internalized Stigma scale  *Berger (short) | sf_stig5 | People's attitudes about HIV make you feel worse about yourself. | 4, Strongly agree  3, Agree  2, Disagree  1, Strongly disagree | |  |
| PLHIV internalized Stigma scale  *Berger (short) | sf_stig6 | You feel guilty because you have HIV. | 4, Strongly agree  3, Agree  2, Disagree  1, Strongly disagree | |  |
| PLHIV internalized Stigma scale  *Berger (short) | sf_stig7 | You feel ashamed of having HIV. | 4, Strongly agree  3, Agree  2, Disagree  1, Strongly disagree | |  |
| PLHIV internalized Stigma scale  *Berger (short) | sf_stig8 | It is easier to avoid friends than to tell them you have HIV. | 4, Strongly agree  3, Agree  2, Disagree  1, Strongly disagree | |  |
| PLHIV internalized Stigma scale | sf_stig9 | You feel completely worthless because you have HIV. | 4, Strongly agree  3, Agree  2, Disagree  1, Strongly disagree | |  |
| PLHIV internalized Stigma scale | sf_stig10 | You feel that you bring a lot of trouble to your family because you have HIV. | 4, Strongly agree  3, Agree  2, Disagree  1, Strongly disagree | |  |
| **Anticipated HIV-Related Stigma** | | | | | |
| PLHIV anticipated stigma scale  *adapted Zelaya | at_stig1 | You are afraid that if you reveal your HIV status to your friends,  they will lose respect for you. | 4, Strongly agree  3, Agree  2, Disagree  1, Strongly disagree | |  |
| PLHIV anticipated stigma scale | at_stig4 | You fear that you could be threatened with violence if your HIV status became publicly known. | 4, Strongly agree  3, Agree  2, Disagree  1, Strongly disagree | |  |
| PLHIV anticipated stigma scale | at_stig5 | You fear that if you disclosed your HIV status to the people you work with, they could fire you or take your clients away. | 4, Strongly agree  3, Agree  2, Disagree  1, Strongly disagree | |  |
| PLHIV anticipated stigma scale | at_stig6 | You fear that your partner would desert you if your HIV status became known. | 4, Strongly agree  3, Agree  2, Disagree  1, Strongly disagree | |  |
| PLHIV anticipated stigma scale | at_stig7 | You fear that your family would exclude you from usual family activities if your HIV status became known. | 4, Strongly agree  3, Agree  2, Disagree  1, Strongly disagree | |  |
| **HIV-Related Stigma and Discrimination** | | | | | |
| PLHIV Enacted Stigma Scale  *Berger (short) | Stigma9 | I have lost friends by telling them I have HIV. | 4, Strongly agree  3, Agree  2, Disagree  1, Strongly disagree | |  |
| PLHIV Enacted Stigma Scale  *Berger (short) | Stigma10 | People I care about stopped speaking to me after learning I had HIV. | 4, Strongly agree  3, Agree  2, Disagree  1, Strongly disagree | |  |
| PLHIV Enacted Stigma Scale  *Berger (short) | Stigma11 | I have lost my job because of having HIV. | 4, Strongly agree  3, Agree  2, Disagree  1, Strongly disagree | |  |
| PLHIV Enacted Stigma Scale  *Berger (short) | Stigma6 | I have lost a romantic partner because of having HIV. | 4, Strongly agree  3, Agree  2, Disagree  1, Strongly disagree | |  |
| PLHIV Enacted Stigma Scale  *Berger (short) | Stigma13 | I have been denied health care because of having HIV. | 4, Strongly agree  3, Agree  2, Disagree  1, Strongly disagree | |  |
| **HIV Care** | | | | | |
| **Statement to read participant before questions:** | | **I am going to ask you if you have received any HIV-related medical care. By medical care, I mean physical exams, lab tests, and treatment for HIV and any other kinds of infections that have come from having HIV.** | | | |
| Treatment and care behaviors | hiv_care | Have you ever received HIV related medical care? | Yes  No |  | |
| Treatment and care behaviors | care_loc | Where do you receive most of your HIV related health care services? (in addition to seeking ART drugs) | Hospital Luis Eduardo Aybar (MORGAN)  Health Center  COIN  IDCP  Lotes y Servicio  Clínica del 20-30  National Laboratory  IDEV  Other (specify)______  Doesn’t know | Show if hiv_care=Yes | |
| Treatment and care behaviors | care_frq_nw | In the past 6 months, how many times did you go to an HIV-related medical appointment (other than looking for ART medication)? | 0 times  1 time  2 times  3 times  4 times  5 times  6 to 10 times  More than 10 times | Show if hiv_care=Yes | |
| Treatment and care behaviors | clinic_frq | In the past 6 months, how often have you gone to SAI to get your HIV medications? | More than once a month.  About once a month  About every two months  About every three months  Between every three to six months  Every 6 months  Less than every six months  Never |  | |
| Treatment and care behaviors | care_missapt | In the past 6 months, how many HIV-related doctor’s appointments have you missed? | 0 times  Once  Twice  Three times  Four times  Five times  6 to 10 times  More than 10 times |  | |
| **Experience with Antiretroviral Therapy (ART)** | | | | | |
| ART Use and Adherence | art_ever | Have you ever taken antiretrovirals (ARTs)? | Yes  No |  | |
| ART Use and Adherence | art_strt | In what year did you start taking antiretrovirals (ARTs) (e.g. 1992, 2010)? | ___ | Show if art_ever=Yes  Min: 1980  Max: 2023 | |
| ART USE and Adherence | art_curr | Are you currently taking any antiretroviral therapy (ART)? | Yes  No |  | |
| ART USE and Adherence | art_evrstop1 | Have you ever stopped or discontinued antiretrovirals (ARTs)? | Yes  No | Show if art_ever=Yes | |
| ART USE and Adherence | art_6mstop1 | In the last 6 months, have you stopped or suspended the antiretrovirals (ART)? | Yes  No | Show if art_curr=Yes | |
| ART USE and Adherence | art_6mstop2 | How many times have you stopped taking antiretroviral (ART) medication in the last 6 months? | ________ times | Show if art_6mstop1=Yes | |
| ART USE and Adherence | art_6mstop3 | For how long did you stop taking antiretrovirals (ARTs) in the last 6 months? | For one day  For one week  For 2-4 weeks  For more than one month | Show if art_6mstop1=Yes | |
| ART USE and Adherence | art_adh1 | During the last 4 days, how many days have you not taken your dose of antiretrovirals (ART)? | 0 days  1 day  2 days  3 days  4 days  Don’t know | Show if art_curr=Yes | |
| ART USE and Adherence | art_adh1a | How many antiretroviral pills (ART) do you take in a day? | ___ | Show if art_curr=Yes  Max 99 | |
| ART USE and Adherence | art_adh2a | Thinking again about the last 4 days, how closely did you follow your specific schedule? | Perfectly  Very closely  Closely  Not very closely |  | |
| ART USE and Adherence | art_adh5 | Some people find that they forget to take their pills on the weekend days. Did you stop taking any of your HIV medications this past weekend? | Yes  No | Show if art_curr==”yes” | |
| ART USE and Adherence | art_adh6 | When was the last time you stopped taking any of your medications? | This week  1 to 2 weeks ago  2 to 4 weeks ago  1 to 3 months ago  More than 3 months ago  I have never stopped taking my medications | Show if art_curr==”yes” | |
| ART USE and Adherence | art_share1 | Have you ever shared your antiretrovirals (ARTs) with anyone? | Yes  No | Show if art_ever=Yes | |
| ART USE and Adherence | art_share2 | Has anyone ever shared their antiretrovirals (ARTs) with you? | Yes  No | Show if art_ever=Yes | |
| **ART-Related Stigma** | | | | | |
| **Statement to read participant before questions:** | | **Please tell me if you agree or disagree with the following statements:** | |  | |
| ART-Related Stigma  *Rintamaki | med_stig1 | I don't want people to see me taking my antiretrovirals (ARTs). | Agree (3)  Not sure (2)  Disagree (1) | Show if art_curr==”yes” | |
| ART-Related Stigma  *Rintamaki | med_stig2 | I am embarrassed to go to the clinic or pharmacy to pick up my antiretrovirals (ARTs). | Agree (3)  Not sure (2)  Disagree (1) | Show if art_curr==”yes” | |
| ART-Related Stigma  *Rintamaki | med_stig3 | Taking my antiretrovirals (ARTs) reminds me that I have HIV. | Agree (3)  Not sure (2)  Disagree (1) | Show if art_curr==”yes” | |
| **HIV Treatment Options** | | | | | |
| **Statement to read participants before questions:** | | **Now I would like to ask you some questions about new HIV treatment options that are under study. One possible option is a weekly pill to treat HIV. If the weekly HIV pill is proven to be as safe and effective as daily HIV pills:** | | | |
|  | artpill | How likely would you be to use a weekly pill to treat HIV if it were available? | 4, Very likely  3, Likely  2, Unlikely  1, Very unlikely |  | |
|  | pillpref | Thinking about your preference between a weekly pill compared to a daily pill to treat HIV. You would have: | 1, Strong preference for taking an HIV pill once a week  2, Some preference for taking an HIV pill once a week  3, Some preference for taking HIV pills once daily  4, Strong preference for taking an HIV pill once a day |  | |
| **Statement to read participants before questions:** | | **In addition to pills, injections to treat HIV are available in some places. These injections are given by a provider at a clinic once a month.** | | | |
|  | arttxoption | In your opinion, which HIV treatment option would you prefer the most, if they all cost the same and work the same: | 1, Take pills once a day  2, Taking one pill once a week  3, Receiving an injection once a month at a clinic |  | |
|  | artpractical | We also want to understand the practical and lifestyle advantages that influence your preferences:  What is the primary practical advantage of the HIV treatment option you preferred? | 1, Mode: pill vs. injection  2, Effectiveness (how well it works)  3, Side effects  4, Time required    5, Other, please specify: |  | |
|  | artlifestyle | What is the primary lifestyle advantage of the HIV treatment option you preferred? | 1, Convenience  2, Privacy  3, Less stress  4, Easier to take as prescribed (easier to take as directed)  5, Other, please specify: |  | |
| **Quality of Care** | | | | | |
| **Statement to read participant before questions:** | | **Now I'm going to ask you some questions about the environment in the clinic where you receive most of your HIV-related care.** | | | |
| Clinic dynamic | qualcare1 | The team at the clinic where you receive HIV care is available to help you... | Always  Most of the time  Some of the time  Rarely or never  Don't know | Show if hiv_care=Yes | |
| Clinic dynamic | qualcare2 | The staff at the clinic where you receive HIV care treats you with respect.... | Always  Most of the time  Some of the time  Rarely or never  Don't know | Show if hiv_care=Yes | |
| Clinic dynamic | qualcare3 | In general, the services at the clinic where you receive HIV care are... | Excellent  Very good  Good  Adequate  Weak  Don't Know | Show if hiv_care=Yes | |
| Provider continuity | qualcare4 | Are you seen by the same provider (health staff) every time you visit the clinic where you receive your HIV care? | Yes  No | Show if hiv_care=Yes | |
| **Communication with the HIV Care Provider** | | | | | |
| **Statement to read participant before questions:** | | **Now I am going to read you some sentences about how you perceive the care you receive for HIV so that you can tell me your level of agreement or disagreement.** | | | |
| Patient-provider communication  * Patient Reactions Assessment (PRA), Galassi | pra_care1 | I understand the side effects of my treatment. | 4, Strongly agree  3, Agree  2, Disagree  1, Strongly disagree | | Show if hiv_care=Yes |
| Patient-provider communication  * Patient Reactions Assessment (PRA), Galassi | pra_care2 | When I talk to HIV care providers, it is difficult to get them to clarify information that I don't understand. | 1, Strongly agree  2, Agree  3, Disagree  4, Strongly disagree | | Show if hiv_care=Yes |
| Patient-provider communication  * Patient Reactions Assessment (PRA), Galassi | pra_care3 | HIV care providers are kind to me. | 4, Strongly agree  3, Agree  2, Disagree  1, Strongly disagree | | Show if hiv_care=Yes |
| Patient-provider communication  * Patient Reactions Assessment (PRA), Galassi | pra_care4 | It's hard to ask HIV care providers about something I don't understand. | 1, Strongly agree  2, Agree  3, Disagree  4, Strongly disagree | | Show if hiv_care=Yes |
| Patient-provider communication  * Patient Reactions Assessment (PRA), Galassi | pra_care6 | Providers make me feel comfortable talking about my personal problems (issues). | 4, Strongly agree  3, Agree  2, Disagree  1, Strongly disagree | | Show if hiv_care=Yes |
| Patient-provider communication* Patient Reactions Assessment (PRA), Galassi | pra_care7 | It is difficult for me to tell providers about new symptoms. | 1, Strongly agree  2, Agree  3, Disagree  4, Strongly disagree | | Show if hiv_care=Yes |
| Patient-provider communication  * Patient Reactions Assessment (PRA), Galassi | pra_care8 | It is difficult for me to ask how my ART treatment is going. | 1, Strongly agree  2, Agree  3, Disagree  4, Strongly disagree | | Show if hiv_care=Yes |
| Patient-provider communication  * Patient Reactions Assessment (PRA), Galassi | pra_care9 | Providers truly respect me. | 4, Strongly agree  3, Agree  2, Disagree  1, Strongly disagree | | Show if hiv_care=Yes |
| Patient-provider communication  * Patient Reactions Assessment (PRA), Galassi | pra_care10 | I understand the care plan selected for me. | 4, Strongly agree  3, Agree  2, Disagree  1, Strongly disagree | | Show if hiv_care=Yes |
| Patient-provider communication  * Patient Reactions Assessment (PRA), Galassi | pra_care11 | I have a good understanding about the expected changes in my health. | 4, Strongly agree  3, Agree  2, Disagree  1, Strongly disagree | | Show if hiv_care=Yes |
| Patient-provider communication  * Patient Reactions Assessment (PRA), Galassi | pra_care12 | Sometimes I feel insulted when I talk to HIV care providers. | 1, Strongly agree  2, Agree  3, Disagree  4, Strongly disagree | | Show if hiv_care=Yes |
| Patient-provider communication  * Patient Reactions Assessment (PRA), Galassi | pra_care13 | I have difficulty asking questions to the providers. | 1, Strongly agree  2, Agree  3, Disagree  4, Strongly disagree | | Show if hiv_care=Yes |
| Patient-provider communication  * Patient Reactions Assessment (PRA), Galassi | pra_care14 | The providers clearly explain things associated with my treatment. | 4, Strongly agree  3, Agree  2, Disagree  1, Strongly disagree | | Show if hiv_care=Yes |
| Patient-provider communication  * Patient Reactions Assessment (PRA), Galassi | pra_care15 | The providers seem to not be interested in me as a person. | 1, Strongly agree  2, Agree  3, Disagree  4, Strongly disagree | | Show if hiv_care=Yes |
| **Cost of HIV Care** | | | | | |
| Mode of travel to clinic | clinic_trvl | How do you get to the clinic for your HIV appointments most of the time? | Public Transportation  Taxi  Private car  On foot  Other (specify) | | Show if hiv_care=Yes |
| Time overall | clinic_ovrall_tm | On days when you have a medical appointment, how long in total do you take from the time you leave home until you return? | ______ Hours  ______ Minutes | | Show if hiv_care=Yes |
| Time to clinic | clinic_tm | How long does it take you to get to the clinic where you receive HIV care? | ______ Hours  ______ Minutes | | Show if hiv_care=Yes |
| Care costs – travel (round-trip) | clinic_cost2 | How much does it cost to travel to and from the SAI where you receive HIV care? | RD$_____ | | Show if hiv_care=Yes  Max: 5000 |
| Care costs – food | clinic_cost3 | Do you spend money on food when you go to an SAI appointment? | Yes  No | | Show if hiv_care=Yes |
| Care costs - clinic fee | clinic_cost9 | Do you pay for your HIV appointment? | Yes No | | Show if hiv_care=Yes |
| Care costs - clinic fee | clinic_cost10 | Normally, how much do you pay for your HIV appointment? | RD$_____ | | Show if clinic_cost9==”yes” |
| Care costs - meds | clinic_cost7 | Do you pay for your HIV medications? | Yes No | | Show if hiv_care=Yes |
| Care costs - meds | clinic_cost8 | How much do you pay for your HIV medications? | RD$_____ | | Show if clinic_cost7=Yes |
| **Knowledge of CD4 and Viral Load** | | | | | |
| **Statement to read participants before the questions:** | | We would like to know if you are familiar with two HIV terms: CD4 and viral load. Now I'm going to ask you some questions about this. | | | |
| Brief Estimate of Health Knowledge and Action—HIV (BEHKA-HIV) | cd4_knwlg | Does ARV treatment serve to increase (raise) or decrease (lower) the CD4 count? | Increase (1)  Decrease (0)  Don't know | |  |
| Brief Estimate of Health Knowledge and Action—HIV (BEHKA-HIV) | viral_knwlg | Does the treatment serve to increase (raise) or decrease (lower) the viral load? | Increase (1)  Decrease (0)  Don't know | |  |

## Section IV: Other Health Conditions

| **Variable Purpose** | **Variable Name** | **Question** | **Response Options** | **Logic** |
| --- | --- | --- | --- | --- |
| **Statement to read participant before questions:** | | Now I would like to ask you some questions about sexually transmitted infections, STIs. Please don't feel shy to answer, as everything you share with me is private and will be treated confidentially. | | |
| STI history | sti2 | In the last 6 months, have you been diagnosed with a sexually transmitted infection other than HIV (such as syphilis, gonorrhea, chlamydia, or herpes)? | Yes  No |  |
| STI history | ti2_spec | Which sexually transmitted infection(s) were you told you had in the past 6 months?  *[Mark all that apply]* | Syphilis  Gonorrhea  Chlamydia  Herpes  Chancroid  Genital warts  Other (specify)_____  Don’t know | Show if sti2=No |
| STI history | sti4 | Have you used medications for the STI? | Yes, indicated by a physician  Yes, self-medicated  No | Show if sti2=No |
| **Other Health Conditions** | | | | |
| **Statement to read participant before questions:** | | **Now, I would like to ask you some questions about other health screenings.** | | |
| Health Screenings | bpscrn_yr | Have you had your blood pressure taken in the last year? | Yes  No |  |
| Health Screenings | Bpscrn_resyr | Have you ever had high blood pressure in the last year? | Yes  No | Show if bpscrn_yr=Yes |
| Health Screenings | bp_treat | Are you currently taking any blood pressure medication? | Yes  No |  |
| Health Screenings | dp_evr | Have you ever been tested to detect diabetes or sugar? | Yes  No |  |
| Health Screenings | db_diag | Have you been diagnosed with diabetes or prediabetes? | Yes No |  |
| Health Screenings | db_when | In what year were you diagnosed with diabetes or prediabetes (e.g., 1998, 2020)? | ____ YYYY | Show if db_diag=Yes |
| Health Screenings | Db_treat | Are you currently taking any medications for diabetes (or sugar)? | Yes  No |  |
| **Diabetes-Related Stress** | | | | |
| **Statement to read participants before questions:** | | **Now I am going to read some affirmations so that you can tell me how you feel about them. I want you to tell me how much trouble they cause in your life.** | | |
| Diabetes distress | dbdistress1 | Feeling mentally exhausted from the constant effort to control diabetes. | 1, It is not a problem  2, It is a small problem 3, It is a somewhat serious problem  4, It is a serious problem  5, It is a very serious problem | Show if db_diag OR db_treat=Yes |
| Diabetes distress | dbdistress2 | Feeling physically exhausted from the constant effort to control diabetes. | 1, It is not a problem  2, It is a small problem 3, It is a somewhat serious problem  4, It is a serious problem  5, It is a very serious problem | Show if db_diag OR db_treat=Yes |
| Diabetes distress | dbdistress3 | Feeling that my doctor doesn't know enough about diabetes and diabetes care | 1, It is not a problem  2, It is a small problem 3, It is a somewhat serious problem  4, It is a serious problem  5, It is a very serious problem | Show if db_diag OR db_treat=Yes |
| Diabetes distress | dbdistress4 | Not feeling confident in my ability to manage my diabetes on a day-to-day basis. | 1, It is not a problem  2, It is a small problem 3, It is a somewhat serious problem  4, It is a serious problem  5, It is a very serious problem | Show if db_diag OR db_treat=Yes |
| Diabetes distress | dbdistress5 | Feeling angry, scared, or depressed when I think about living with diabetes. | 1, It is not a problem  2, It is a small problem 3, It is a somewhat serious problem  4, It is a serious problem  5, It is a very serious problem | Show if db_diag OR db_treat=Yes |
| Diabetes distress | dbdistress6 | Feeling that my doctor is not giving me specific enough recommendations to manage my diabetes. | 1, It is not a problem  2, It is a small problem 3, It is a somewhat serious problem  4, It is a serious problem  5, It is a very serious problem | Show if db_diag OR db_treat=Yes |
| Diabetes distress | dbdistress7 | Feeling that I am not testing my blood sugar often enough. | 1, It is not a problem  2, It is a small problem 3, It is a somewhat serious problem  4, It is a serious problem  5, It is a very serious problem | Show if db_diag OR db_treat=Yes |
| Diabetes distress | dbdistress8 | Feeling that no matter what I do, I will always have serious long-term complications. | 1, It is not a problem  2, It is a small problem 3, It is a somewhat serious problem  4, It is a serious problem  5, It is a very serious problem | Show if db_diag OR db_treat=Yes |
| Diabetes distress | dbdistress9 | Feeling that I often fail to take control of my diabetes. | 1, It is not a problem  2, It is a small problem 3, It is a somewhat serious problem  4, It is a serious problem  5, It is a very serious problem | Show if db_diag OR db_treat=Yes |
| Diabetes distress | dbdistress10 | Feeling that neither my friends nor my family give me enough support in my efforts to take care of myself (e.g., planning activities that clash with my schedule, encouraging me to eat unhealthy foods). | 1, It is not a problem  2, It is a small problem 3, It is a somewhat serious problem  4, It is a serious problem  5, It is a very serious problem | Show if db_diag OR db_treat=Yes |
| Diabetes distress | dbdistress11 | Feeling that diabetes controls my life. | 1, It is not a problem  2, It is a small problem 3, It is a somewhat serious problem  4, It is a serious problem  5, It is a very serious problem | Show if db_diag OR db_treat=Yes |
| Diabetes distress | dbdistress12 | Feeling that my doctor does not take my concerns seriously. | 1, It is not a problem  2, It is a small problem 3, It is a somewhat serious problem  4, It is a serious problem  5, It is a very serious problem | Show if db_diag OR db_treat=Yes |
| Diabetes distress | dbdistress13 | Feeling that I am not following the recommended diet. | 1, It is not a problem  2, It is a small problem 3, It is a somewhat serious problem  4, It is a serious problem  5, It is a very serious problem | Show if db_diag OR db_treat=Yes |
| Diabetes distress | dbdistress14 | Feeling that my friends don't know how hard it is to live with diabetes. | 1, It is not a problem  2, It is a small problem 3, It is a somewhat serious problem  4, It is a serious problem  5, It is a very serious problem | Show if db_diag OR db_treat=Yes |
| Diabetes distress | dbdistress15 | Feeling that my family doesn't know how hard it is to live with diabetes. | 1, It is not a problem  2, It is a small problem 3, It is a somewhat serious problem  4, It is a serious problem  5, It is a very serious problem | Show if db_diag OR db_treat=Yes |
| Diabetes distress | dbdistress16 | Feeling overwhelmed by the care it takes to live with diabetes. | 1, It is not a problem  2, It is a small problem 3, It is a somewhat serious problem  4, It is a serious problem  5, It is a very serious problem | Show if db_diag OR db_treat=Yes |
| Diabetes distress | dbdistress17 | Feeling like I don't have a doctor I can see often enough to discuss my diabetes. | 1, It is not a problem  2, It is a small problem 3, It is a somewhat serious problem  4, It is a serious problem  5, It is a very serious problem | Show if db_diag OR db_treat=Yes |
| Diabetes distress | dbdistress18 | Feeling that I don't have the motivation to manage my diabetes. | 1, It is not a problem  2, It is a small problem 3, It is a somewhat serious problem  4, It is a serious problem  5, It is a very serious problem | Show if db_diag OR db_treat=Yes |
| Diabetes distress | dbdistress19 | Feeling that neither my friends nor my family give me the emotional support I would like to have. | 1, It is not a problem  2, It is a small problem 3, It is a somewhat serious problem  4, It is a serious problem  5, It is a very serious problem | Show if db_diag OR db_treat=Yes |
| Statement to read to participants: | | Now I am going to read some statements about access to food. I want you to tell me how often they occur. | | |
|  | foodsec1 | The food I bought did not yield enough, and I did not have money to buy more. Did this happen often, sometimes, or never in the last 12 months? | 3, Frequently  2, Sometimes  1, Never  0, Don't know, did not answer |  |
|  | foodsec2 | The food I bought did not yield enough, and I had no money to buy more. Did this happen often, sometimes, or never in the last 12 months? | 3, Frequently  2, Sometimes  1, Never  0, Don't know, did not answer |  |

## Section V: Mental Health

| **Variable Purpose** | **Variable Name** | **Question** | **Response Options** | **Logic** |
| --- | --- | --- | --- | --- |
| **Anxiety** | | | | |
| **Statement to read participant before questions:** | | **Now I am going to ask you some questions about how you have been feeling in the last 2 weeks.**  **Tell me the first thing that comes to your mind; your immediate response to these questions is better.**  **Please tell me which answer is closest to your experience:** | | |
| Anxiety -  Hospital Anxiety and Depression Scale (HADS) | hads_anx1 | I feel tense or 'wound up' | 3 Every day  2 A lot of the time  1 Sometimes  0 Never |  |
| Anxiety -  Hospital Anxiety and Depression Scale (HADS) | hads_anx2 | I get a sort of strange sensation, like “butterflies” in the stomach. | 0 Never  1 in certain occasions  2 Quite often  3 Very often | *Note reverse coding |
| Anxiety -  Hospital Anxiety and Depression Scale (HADS) | hads_anx3 | I get a sort of frightened feeling as if something awful is about to happen. | 3 Very definitely and quite badly  2 Yes, but not too badly  1 A little, but it doesn’t worry me  0 Not at all |  |
| Anxiety -  Hospital Anxiety and Depression Scale (HADS) | hads_anx4 | I feel restless as if I have to be on the move. | 3 Very much  2 Quite a lot  1 Not much  0 Not at all |  |
| Anxiety -  Hospital Anxiety and Depression Scale (HADS) | hads_anx5 | Worrying thoughts go through my mind. | 3 Majority of the times  2 A lot of the time  1 Someitmes, but not too often  0 Only occasionally |  |
| Anxiety -  Hospital Anxiety and Depression Scale (HADS) | hads_anx6 | I get sudden feelings of panic. | 3 Very often  2 Quite often  1 Not very often  0 Rarely |  |
| Anxiety -  Hospital Anxiety and Depression Scale (HADS) | hads_anx7 | I can sit at ease and feel relaxed. | 0 Always  1 Generally  2 Not very often  3 Never | *Note reverse coding |
| **Depression** | | | | |
| **Statement to read participant before questions:** | | **Now I'm going to ask you some questions if any of the following have bothered you in the last 2 weeks....** | | |
| Mental Health (PHQ-9) | phq9_mh1 | Little interest or pleasure in doing things | Not at all  Several days  More than one half of the days  Nearly every day |  |
| Mental Health (PHQ-9) | phq9_mh2 | Feeling down, depressed, or hopeless | Not at all  Several days  More than one half of the days  Nearly every day |  |
| Mental Health (PHQ-9) | phq9_mh3 | Trouble falling asleep, staying asleep, or sleeping too much | Not at all  Several days  More than one half of the days  Nearly every day |  |
| Mental Health (PHQ-9) | phq9_mh4 | Feeling tired or having little energy | Not at all  Several days  More than one half of the days  Nearly every day |  |
| Mental Health (PHQ-9) | phq9_mh5 | Poor appetite or overeating | Not at all  Several days  More than one half of the days  Nearly every day |  |
| Mental Health (PHQ-9) | phq9_mh6 | Feeling bad about yourself – or that you’re a failure or have let yourself or your family down | Not at all  Several days  More than one half of the days  Nearly every day |  |
| Mental Health (PHQ-9) | phq9_mh7 | Trouble concentrating on things, such as reading the newspaper or watching television | Not at all  Several days  More than one half of the days  Nearly every day |  |
| Mental Health (PHQ-9) | phq9_mh8 | Moving or speaking so slowly that other people could have noticed; or the opposite – being so fidgety or restless that you have been moving around a lot more than usual | Not at all  Several days  More than one half of the days  Nearly every day |  |
| Mental Health (PHQ-9) | phq9_mh10 | If you have experienced any of the problems just listed, how difficult have those problems made it for you to do your work, take care of things at home, or get along with other people? | Not difficult at all  Somewhat difficult  Very difficult  Extremely difficult |  |
| Mental Health (PHQ-9) | phq9_mh9 | Thoughts that you would be better off dead or of hurting yourself in some way | Not at all  Several days  More than one half of the days  Nearly every day |  |
| **Suicide** | | | | |
| **Variable purpose** | **Variable name** | **Question** | **Response Options** | **Logic** |
| **Statement to read participant before questions:** | | **Now I am going to ask you some questions about the thoughts we could have about our future and life.** | | |
| Mental Health (suicidal ideation) | SI_1 | Have you ever thought about ending your life? | Yes  No |  |
| Mental Health (suicidal ideation) | SI_2 | Have you thought about ending your life in the last 6 months? | Yes  No | Show if si_1 is Yes |
| Mental Health (suicide attempt) | SA_1 | Have you ever tried to end your life? | Yes  No |  |
| Mental Health (suicidal attempt) | SA_2 | Have you tried to end your life in the last 6 months? | Yes  No | Show if sa_1=Yes |
| **Mindfulness** | | | | |
| **Statement to read participant before questions:** | | **I am now going to read you some statements and I’d like you to tell me how often you feel these statements describe your thoughts or feelings.** | | |
| Cognitive and Affective Mindfulness Scale-Revised | CAMS-R1 | It is easy for me to concentrate on what I am doing. | (1) Rarely/Not at All  (2) Sometimes  (3) Often  (4) Almost Always |  |
| Cognitive and Affective Mindfulness Scale-Revised | CAMS-R3 | I can tolerate emotional pain. | (1) Rarely/Not at All  (2) Sometimes  (3) Often  (4) Almost Always |  |
| Cognitive and Affective Mindfulness Scale-Revised | CAMS-R4 | I can accept things I cannot change. | (1) Rarely/Not at All  (2) Sometimes  (3) Often  (4) Almost Always |  |
| Cognitive and Affective Mindfulness Scale-Revised | CAMS-R5 | I can usually describe how I feel at the moment in considerable detail. | (1) Rarely/Not at All  (2) Sometimes  (3) Often  (4) Almost Always |  |
| Cognitive and Affective Mindfulness Scale-Revised | CAMS-R6 | I am easily distracted. | (4) Rarely/Not at All  (3) Sometimes  (2) Often  (1) Almost Always | *Note reversed coding |
| Cognitive and Affective Mindfulness Scale-Revised | CAMS-R8 | It’s easy for me to keep track of my thoughts and feelings. | (1) Rarely/Not at All  (2) Sometimes  (3) Often  (4) Almost Always |  |
| Cognitive and Affective Mindfulness Scale-Revised | CAMS-R9 | I try to notice (or recognize) my thoughts without judging them. | (1) Rarely/Not at All  (2) Sometimes  (3) Often  (4) Almost Always |  |
| Cognitive and Affective Mindfulness Scale-Revised | CAMS-R10 | I am able to accept the thoughts and feelings I have. | (1) Rarely/Not at All  (2) Sometimes  (3) Often  (4) Almost Always |  |
| Cognitive and Affective Mindfulness Scale-Revised | CAMS-R11 | I am able to focus on the present moment. | (1) Rarely/Not at All  (2) Sometimes  (3) Often  (4) Almost Always |  |
| Cognitive and Affective Mindfulness Scale-Revised | CAMS-R12 | I am able to pay close attention to one thing for a long period of time. | (1) Rarely/Not at All  (2) Sometimes  (3) Often  (4) Almost Always |  |
| **Self-esteem** | | | | |
| **Statement to read participant before questions:** | | **Now I want you to think about how you feel and think about yourself. I will read you a series of sentences and I want you to tell me your level of agreement or disagreement.** | | |
| Rosenberg | AE_1 | In general, I am satisfied with myself | 4, Totally agree  3, Agree  2, Disagree  1, Strongly Disagree |  |
| Rosenberg | AE_2 | Sometimes I think I'm not good at anything | 1, Totally agree  2, Agree  3, Disagree  4, Strongly Disagree |  |
| Rosenberg | AE_3 | I have the feeling that I possess some good qualities | 4, Totally agree  3, Agree  2, Disagree  1, Strongly Disagree |  |
| Rosenberg | AE_4 | I am able to do things as well as most people are able to do | 4, Totally agree  3, Agree  2, Disagree  1, Strongly Disagree |  |
| Rosenberg | AE_5 | I feel like I don't have too many things to be proud of | 1, Totally agree  2, Agree  3, Disagree  4, Strongly Disagree |  |
| Rosenberg | AE_6 | Sometimes I feel really useless | 1, Totally agree  2, Agree  3, Disagree  4, Strongly Disagree |  |
| Rosenberg | AE_7 | I have the feeling that I am worth at least as much as  the same as most people | 4, Totally agree  3, Agree  2, Disagree  1, Strongly Disagree |  |
| Rosenberg | AE_8 | I wish I respected myself more | 1, Totally agree  2, Agree  3, Disagree  4, Strongly Disagree |  |
| Rosenberg | AE_9 | In short, I think I am a failure | 1, Totally agree  2, Agree  3, Disagree  4, Strongly Disagree |  |
| Rosenberg | AE_10 | I have a positive attitude towards myself | 4, Totally agree  3, Agree  2, Disagree  1, Strongly Disagree |  |
| **Resilience** | | | | |
| **Statement to read participants before questions:** | | **Now I want you to think about how you see and solve difficult situations in your life. I will read you a series of sentences and I want you to tell me your level of agreement or disagreement.** | | |
| Resiliencia  (Brief Resilience Scale) | RES_1 | I bounce back quickly after going through difficult situations. | 5 Strongly agree  4, Agree  3, Neither agree nor disagree  2, Disagree  1 Strongly disagree |  |
| Resiliencia  (Brief Resilience Scale) | RES_3 | It doesn't take me long to recover from a stressful event. | 5 Strongly agree  4 Agree  3 Neither agree nor disagree  2 Disagree  1 Strongly disagree |  |
| Resiliencia  (Brief Resilience Scale) | RES_5 | I usually overcome difficult situations with little difficulty. | 5 Strongly agree  4 Agree  3 Neither agree nor disagree  2 Disagree  1 Strongly disagree |  |

## Section VI: Substance Use

| **Variable Purpose** | **Variable Name** | **Question** | | **Response Options** | **Logic** |
| --- | --- | --- | --- | --- | --- |
| **Alcohol Use** | | | | | |
| **Statement to read participant before questions:** | | **I’d like to ask you questions about drinking alcohol. I know that these are sensitive questions, and that they may not be relevant for you. Please don't feel shy to respond, as everything you share with me is private and will be treated confidentially.** | | | |
| Substance Use  *AUDIT | alc_fq | How often do you drink alcoholic beverages? | Never  Once a month or less  2 to 4 times a month  2 to 3 times a week  4 or more times a week | | If alc_fq=Never  Skip to section on recreational drug use |
| Substance Use  *AUDIT | alc_no | How many drinks do you consume in a typical day when you are drinking? | 1 or 2  3 or 4  5 or 6  7, 8, or 9  10 or more | |  |
| Substance Use  *AUDIT | alc_intx | How often do you have six or more drinks on one occasion? | Never  Less than monthly  Monthly  Weekly  Daily or almost daily | |  |
| Substance Use | alcwhat | On occasions when you drink, what do you normally drink?  *Mark all that apply* | Beer  Rum, whisky or other hard alcohol  Other | |  |
| **Recreational Drug Use** | | | | | |
| **Statement to read participant before questions:** | | **Now I’m going to ask you questions about recreational drug use. I understand that these questions may not be relevant for you.** | | | |
| Substance Use | marj_evr | Have you ever used marijuana? | | Yes  No |  |
| Substance Use | marj_6m | Have you used marijuana in the past 6 months? | | Yes  No | Show if marj_evr=Yes |
| Substance Use | crk_evr | Have you ever used crack (rocks)? | | Yes  No |  |
| Substance Use | crk _6m | Have you used crack (rocks) in the past 6 months? | | Yes  No | Show if crk_evr-Yes |
| Substance Use | coc_evr | Have you ever used cocaine? | | Yes  No |  |
| Substance Use | coc_6m | Have you used cocaine in the past 6 months? | | Yes  No | Show if coc_evr=Yes |
| Substance Use | heroi_evr | Have you ever self-injected drugs in your life? | | Yes  No |  |
| Substance Use | heroi_6m | Have you used self-injected drugs in the past 6 months? | | Yes  No | Show if heroi_evr=Yes |
| Substance Use | ecs_evr | Have you ever used pills (ecstasy)? | | Yes  No |  |
| Substance Use | ecs_6m | Have you used pills (ecstasy) in the past 6 months? | | Yes  No | Show if ecs_evr=Yes |
| Substance Use | od_6m | Have you used any other type of drug in the past 6 months? | | Yes  No |  |
| Substance Use | od_type | What other type of drug have you used? | | Specify: ___________ | Show if od_6m=”Yes” |
| Substance Use | sub_frq | In the last 6 months, how often did you use any of these substance(s)? | | Every day  Once or twice a week (weekly  Once or twice a month (monthly)  A few times in last 6 months  No drugs in the last 6 months |  |
| Substance Use | sub_smokeever | Have you ever smoked cigarettes? | | Yes  No |  |
| Substance Use | sub_smokecurr | Do you currently smoke cigarettes? | | Yes  No | Show if sub_smokeever=Yes |
| Substance Use | sub_vape_ever | Have you ever used vape (electronic cigarette)? | | Yes  No |  |
| Substance Use | sub_vape_6m | Do you currently use vape? | | Yes  No | Show if sub_vape_ever=Yes |
| Substance Use | sub_hook_ever | Have you ever used hookah? | | Yes  No |  |
| Substance Use | hook_6m | Have you used hookah in the last 6 months? | | Yes  No | Show if hook_evr=Yes |

## Section VII: Sex Work

| **Variable Purpose** | **Variable Name** | **Question** | **Response Options** | **Logic** |
| --- | --- | --- | --- | --- |
| **Sex Work History** | | | | |
| **Statement to read participant before questions:** | | **Now I am going to ask you questions about sex work. Please do not feel shy about answering, as everything that you share with me is private and will be treated confidentially.** | | |
| Sex Work History & Environment | agefrstsw | How old were you when you started trading sex for money? | ______ in years | Integer  Min 1  Max 99 |
| Sex Work History & Environment | Sw6m | Have you done sex work in the last 6 months? | Yes  No |  |
| **Sex Work Stigma** | | | | |
| **Statement to read participant before questions:** | | **I would like to know how you speak about your experience with sex work with people around you. Thinking about the last 6 months, for each phrase, I would like to know if it is something that you have done ALWAYS, SOMETIMES, or NEVER:** | | |
| New sex work stigma: Silence | swstig_sil1 | You have tried to make sure that no one knows that you do sex work | 3 Always  2 Sometimes  1 Never |  |
| New sex work stigma: Silence | swstig_sil2 | You have done everything you can to keep sex work a secret | 3 Always  2 Sometimes  1 Never |  |
| New sex work stigma: Silence | swstig_sil3 | You have avoided talking about sex work | 3 Always  2 Sometimes  1 Never |  |
| New sex work stigma: Silence | swstig_sil4 | You have hidden from your family that you do sex work | 3 Always  2 Sometimes  1 Never |  |
| New sex work stigma: Silence | swstig_sil5 | You have ensured that no one in your community finds out about your sex work | 3 Always  2 Sometimes  1 Never |  |
| New sex work stigma: Silence | swstig_sil6 | You have talked to your friends about sex work | 1 Always  2 Sometimes  3 Never |  |
| New sex work stigma: Silence | swstig_sil7 | You have denied that you have worked as a sex worker | 3 Always  2 Sometimes  1 Never |  |
| New sex work stigma: Silence | swstig_sil8 | You have talked about sex work when someone asked you about it | 1 Always  2 Sometimes  3 Never |  |
| **Statement to read participant before questions:** | | **Now, I would like to know how you feel about sex work. Thinking about the last 6 months, for each phrase, I would like to know if you have you felt like this ALWAYS, SOMETIMES or NEVER:** | | |
| New sex work stigma: Shame | swstig_sha1 | Ashamed | 3 Always  2 Sometimes  1 Never |  |
| New sex work stigma: Shame | swstig_sha2 | Valued | 1 Always  2 Sometimes  3 Never |  |
| New sex work stigma: Shame | swstig_sha3 | Comfortable | 1 Always  2 Sometimes  3 Never |  |
| New sex work stigma: Shame | swstig_sha4 | Proud | 1 Always  2 Sometimes  3 Never |  |
| New sex work stigma: Shame | swstig_sha5 | Rejected | 3 Always  2 Sometimes  1 Never |  |
| New sex work stigma: Shame | swstig_sha6 | Accepted | 1 Always  2 Sometimes  3 Never |  |
| New sex work stigma: Shame | swstig_sha7 | At Peace | 1 Always  2 Sometimes  3 Never |  |
| New sex work stigma: Shame | swstig_sha8 | Excluded | 3 Always  2 Sometimes  1 Never |  |
| New sex work stigma: Shame | swstig_sha9 | Different | 3 Always  2 Sometimes  1 Never |  |
| New sex work stigma: Shame | swstig_sha10 | Humiliated | 3 Always  2 Sometimes  1 Never |  |
| New sex work stigma: Shame | swstig_sha11 | Happy | 1 Always  2 Sometimes  3 Never |  |
| New sex work stigma: Shame | swstig_sha12 | Frustrated | 3 Always  2 Sometimes  1 Never |  |
| **Statement to read participant before questions:** | | **Lastly, when thinking about the last 6 months, have people around you done the following things because of your sex work. Please tell me if it is something that has been done to you ALWAYS, SOMETIMES, or NEVER:** | | |
| New sex work stigma: Treatment | swstig_trt1 | Distanced themselves from you | 3 Always  2 Sometimes  1 Never |  |
| New sex work stigma: Treatment | swstig_trt2 | Criticized you | 3 Always  2 Sometimes  1 Never |  |
| New sex work stigma: Treatment | swstig_trt3 | Excluded you from groups | 3 Always  2 Sometimes  1 Never |  |
| New sex work stigma: Treatment | swstig_trt4 | Treated you well | 1 Always  2 Sometimes  3 Never |  |
| New sex work stigma: Treatment | swstig_trt5 | Have not greeted you | 3 Always  2 Sometimes  1 Never |  |
| New sex work stigma: Treatment | swstig_trt6 | Humiliated you | 3 Always  2 Sometimes  1 Never |  |
| New sex work stigma: Treatment | swstig_trt7 | Laughed at you | 3 Always  2 Sometimes  1 Never |  |
| New sex work stigma: Treatment | swstig_trt8 | Have treated you with respect | 1 Always  2 Sometimes  3 Never |  |
| New sex work stigma: Treatment | swstig_trt9 | Called you names | 3 Always  2 Sometimes  1 Never |  |
| New sex work stigma: Treatment | swstig_trt10 | Ignored you | 3 Always  2 Sometimes  1 Never |  |
| New sex work stigma: Treatment | swstig_trt11 | Mistreated you | 3 Always  2 Sometimes  1 Never |  |
| New sex work stigma: Treatment | swstig_trt12 | Treated you differently from other women | 3 Always  2 Sometimes  1 Never |  |

## Section VIII: Sexual Partners

| **Variable Purpose** | **Variable Name** | **Question** | **Response Options** | **Logic** |
| --- | --- | --- | --- | --- |
| **Clients** | | | | |
| **Statement to read participant before questions:** | | **For the next questions, please think about clients who give you money, favors, gifts or services in exchange for sex.** | | |
|  | clnt6m | In the last 6 months have you had sex work clients? | 1, Yes  0, No |  |
| Clients | Clntswk | Normally, how many clients do you have per week? | ______# | Show if clnt6m=Yes  If the answer is 0, skip to next section |
| Clients | meetclnt | In general, where do you meet clients the most?   [Mark all that apply] | Restaurant  Bars/clubs  Hotel/guesthouse  Private parties  Street/park/outside  Mobile phone  Colmadones  Drink (liquor store)  Car wash  Friend(s)  Other (specify)_____ | Show if clnt6m=Yes |
| Clients | internt1 | In the last 6 months, have you used the internet to find or reunite with clients? | Yes  No | Show if clnt6m=Yes |
| Clients | clnt4 | In the last 30 days, how often did you use condoms when you had **vaginal or anal sex** with clients?    We define 'condom use' as using a condom during the entire act of penetrative sex, from beginning to end. | Never  Rarely  Sometimes  Most of the time  Always  Do not perform anal sex | Show if clnt6m=Yes |
| Clients | clnt6 | In the last 30 days, how often did you use condoms when you had **oral sex** (passive or active) with clients? | Never  Rarely  Sometimes  Most of the time  Always  Do not perform oral sex | Show if clnt6m=Yes |
| Clients | clnt_vol | In the last 6 months, has any of your clients hit, slapped, kicked, pushed, or physically hurt you? | Yes  No | Show if clnt6m=Yes |
| Clients | clnt_frcdsx | In the last 6 months, did any of your clients force you to have sex when you did not want to? | Yes  No | Show if clnt6m=Yes |
| Clients | Clnt_viol_hip1 | In the last 6 months, have you told anyone about physical and/or sexual violence that has happened to you with a client? | Yes  No | Show if clnt_viol or clnt_frcdsk=Yes |
| Clients | Clnt_vio_hip2 | Who did you tell?  *[Mark all that apply]* | Health personnel  Navigator  Police  Friend  Family member (relative)  Other (specify)_____ | Show if clnt_viol_hlp1=Yes |
| **Steady/Regular Partners** | | | | |
| **Statement to read participant before questions:** | | **Now I am going to ask you about your fixed partners. Fixed partners are those whom you have had sex with at least three times in your life and who do not pay for sex acts, although they may give you money in general.** | | |
|  | permprtnr | In the last 6 months you have had a fixed partner | Yes  No |  |
| Partner | permprtnr1 | In the last 6 months, how many fixed partners have you had? | ______# | Show if permprtnr=Yes  Interger  Min=1 |
| Partner | permprtnr4 | In the last 30 days, how often did you use condoms when having vaginal or **anal sex** with fixed partners? | Never  Rarely  Sometimes  Almost always  Always  Doesn’t have anal sex | Show if permprtnr=Yes |
| Partner | permprtnr6 | In the last 30 days, how often did you use condoms when giving **oral sex** to fixed partners? | Never  Rarely  Sometimes  Almost always  Always  Doesn't have oral sex | Show if permprtnr=Yes |
| Partner | permpatnr_viol | In the past 6 months, have any of your fixed partners hit, slapped, kicked, pushed, shoved or otherwise physically hurt you? | Yes  No | Show if permprtnr=Yes |
| Partner | permpatnr_frcdsx | In the past 6 months, have any of your fixed partners forced you to have sexual intercourse when you did not want to? | Yes  No | Show if permprtnr=Yes |
| Partner | permpatnr_viol_hlp1 | In the past 6 months, have you told anyone about physical and/or sexual violence that has happened to you with a FIXED PARTNER? | Yes  No | Show if permprtnr_viol or permpatnr_frcdsx=Yes |
| Partner | permpatnr_viol_hlp2 | Who did you tell?  *[Mark all that apply]* | Medical personnel  Navigator  Police  Friend  Family member (relative)  Other (specify)_____ | Show if permprtnr_viol_hlp1=Yes |

## Section IX: Social Cohesion, Social Participation, & Resilience

| **Variable Purpose** | **Variable Name** | **Question** | **Response Options** | **Logic** |
| --- | --- | --- | --- | --- |
| **Social Network** | | | | |
| **Statement to read participant before questions:** | | **Now I would like to talk a little bit about your social network and the important people in your life.** | | |
| **Support Network** | personaimpot | Who is the most important person in your life? | Mother  Father  Sibiling  Niece/nephew  Child  Friend  Other (specify): |  |
| **Statement to read to participants before questions:** | | **Now let's talk a little bit about your relationship with that most important person.  Please, for each sentence, I would like to know if it is something [the most important person] does ALWAYS, SOMETIMES, or NEVER:** | | |
| **Support network** | emsupport | Gives me emotional support | 3 Always  2 Sometimes  1 Never |  |
| **Support network** | ecsupoort | Gives me financial support | 3 Always  2 Sometimes  1 Never |  |
| **Support network** | acceptme | Accept my trans identity.    (or, accept me for who I am?). | 3 Always  2 Sometimes  1 Never |  |
| **Social Support** | | | | |
| **Statement to read participant before questions:** | | **Now I'd like to talk a little bit about your support network. I'm going to read a few sentences and I want you to tell me how often you rely on each type of support.** | | |
| **MOS-SS** | MOS_SS1 | Someone to help you when you can't get out of bed because you are sick. | 3 Always  2 Sometimes  1 Never |  |
| **MOS-SS** | MOS_SS2 | Someone you can count on when you need to talk | 3 Always  2 Sometimes  1 Never |  |
| **MOS-SS** | MOS_SS3 | Someone who can give you good advice about a crisis. | 3 Always  2 Sometimes  1 Never |  |
| **MOS-SS** | MOS_SS4 | Alguien que te lleve al médico cuando lo necesita | 3 Always  2 Sometimes  1 Never |  |
| **MOS-SS** | MOS_SS5 | Someone to take you to the doctor when needed | 3 Always  2 Sometimes  1 Never |  |
| **MOS-SS** | MOS_SS6 | Someone to have a good time with | 3 Always  2 Sometimes  1 Never |  |
| **MOS-SS** | MOS_SS7 | Someone to inform you and help you understand a situation. | 3 Always  2 Sometimes  1 Never |  |
| **MOS-SS** | MOS_SS8 | Someone to confide in or talk to about yourself and your concerns | 3 Always  2 Sometimes  1 Never |  |
| **Social Cohesion** | | | | |
| **Statement to read participant before questions:** | | **Now I want you to think about your relationships with other trans women. I will read you a series of sentences and I want you to tell me your level of agreement or disagreement.** | | |
| Social Cohesion | soccoh01 | You can count on the trans women you know if you need to borrow money. | 4, Strongly agree  3, Agree  2, Disagree  1, Strongly Disagree |  |
| Social Cohesion | Soccoh02 | You can count on the trans women you know to accompany you to the doctor or hospital. | 4, Strongly agree  3, Agree  2, Disagree  1, Strongly Disagree |  |
| Social Cohesion | soccoh03 | You can count on the trans women you know if you need to talk about your problems. | 4, Strongly agree  3, Agree  2, Disagree  1, Strongly Disagree |  |
| Social Cohesion | soccoh04 | In general, the trans women you know only care about themselves. | 1, Strongly agree  2, Agree  3, Disagree  4, Strongly Disagree |  |
| Social Cohesion | soccoh05 | You can count on the trans women you know if you need a place to stay. | 4, Strongly agree  3, Agree  2, Disagree  1, Strongly Disagree |  |
| Social Cohesion | soccoh06 | You can count on the trans women you know to help you with a violent or difficult client. | 4, Strongly agree  3, Agree  2, Disagree  1, Strongly Disagree |  |
| Social Cohesion | soccoh07 | You can count on the trans women you know to help you find sex work clients. | 4, Strongly agree  3, Agree  2, Disagree  1, Strongly Disagree |  |
| Social Cohesion | soccoh08 | You can count on the trans women you know to support your decision to use condoms. | 4, Strongly agree  3, Agree  2, Disagree  1, Strongly Disagree |  |
| Social Cohesion | soccoh09 | The trans women you know function as an integrated group. | 4, Strongly agree  3, Agree  2, Disagree  1, Strongly Disagree |  |
| Social Cohesion | soccoh10 | In general the trans women you know are always arguing with each other. | 1, Strongly agree  2, Agree  3, Disagree  4, Strongly Disagree |  |
| Social Cohesion | soccoh11 | You can trust most of the women sex workers you know. | 4, Strongly agree  3, Agree  2, Disagree  1, Strongly Disagree |  |
| **Social Participation** | | | | |
| **Statement to read participant before questions:** | | **There are different groups of which you can be part of. Please tell me if you consider yourself a member of the following groups:** | | |
| Social Participation | socgrp01 | Church or religious groups | Yes  No |  |
| Social Participation | socgrp02 | Clubs (sports, students, association of fathers, mothers and friends of the school, recreational, social) | Yes  No |  |
| Social Participation | socgrp03 | Cultural activities (dance, music, etc.) | Yes  No |  |
| Social Participation | socgrp04 | Neighborhood meetings (neighborhood association) | Yes  No |  |
| Exposure to Intervention Activities | expsr24 | In the last 6 months, have you attended a workshop at an organization that works with women sex workers such as COTRAVED or TRANSSA? | Yes  No |  |
| Exposure to Intervention Activities | expsr25 | What was the main theme of the workshop? | Trans rights  HIV  Financial security  Hormones  Violence  Other_____ | Show if expsr24=Yes |
| Social Participation | socgrp07 | In the past 6 months, how often have you participated in an activity, march, or protest to promote the human rights of women sex workers? | Never  Rarely  A couple of times Frequently |  |
| Social Participation | socgrp08 | In the past 6 months, how often have you gotten together with other women sex workers to speak with government officials or political leaders to address the problems that women sex workers face? | Never  Rarely  A couple of times Frequently |  |
| Social Participation | socgrp09 | In the past 6 months, how often have you joined together with other women sex workers to address common problems that women sex workers face? | Never  Rarely  A couple of times Frequently |  |
| Social Participation | socgrp10 | In the past 6 months, how often have you participated in an organized group with other trans women? | Never  Rarely  A couple of times Frequently |  |
| **Social Inclusion** | | | | |
| **Statement to read participant before questions:** | | Now I am going to detail some official documents and I want you to tell me if you have them or not. | | |
| Social Inclusion | soc_inc1a | Do you have an identity and voter registration card? | Yes  No |  |
| Social Inclusion | soc-inc1b | Do you have a birth certificate? | Yes  No |  |
| Social Inclusion | soc_inc2 | Do you have a bank account*?* | Yes  No |  |
| Social Inclusion | soc_inc2a | Do you have health insurance? | Yes  No |  |

## Section X: Exposure to Interventions

| **Variable Purpose** | **Variable Name** | **Questions** | **Response Options** | **Logic** |
| --- | --- | --- | --- | --- |
| **Statement to read participant before questions:** | | **Now I am going to ask you some questions about your participation to HIV-related activities and the trans community.** | | |
| Exposure to Intervention Activities | expsr07 | In the past 6 months, have you had contact with a peer educator/navigator, that is – someone that provided support and helped you access services? | Yes  No  Unsure |  |
| Exposure to Intervention Activities | expsr07_phn | How many times have you had contact with this person through a phone call in the past 6 months? | __ | Show if expsr07=Yes  Integer  Max: 99 |
| Exposure to Intervention Activities | expsr07_txt | How many times have you had contact with this person through text messages in the past 6 months? | __ | Show if expsr07=Yes  Integer  Max: 99 |
| Exposure to Intervention Activities | expsr07_prsn | How many times have you had in-person contact (face to face) with this person in the past 6 months? | __ | Show if expsr07=Yes  Integer  Max: 99 |
| Exposure to Intervention Activities | expsr_pn1 | Have you been reminded of medical (health) appointments by this person in the past 6 months? | Yes  No | Show if expsr07=Yes |
| Exposure to Intervention Activities | expsr_pn2 | Were you accompanied by this person to SAI appointments in the past 6 months? | Yes  No | Show if expsr07=Yes |
| Exposure to Intervention Activities | expsr_othera | Has this person accompanied you to any other appointments related to health in the past 6 months? | Yes  No | Show if expsr07=Yes |
| Exposure to Intervention Activities | expsr_othera2 | Has this person accompanied you to any other appointments that were NOT health related in the last 6 months? |  | Show if expsr07=Yes |
| Exposure to Intervention Activities | expsr_otherb | To what type of appointments (not related to health) did this person accompany you to? | Legal services  School appointments  Work appointments  Other type of appointment (specify) | Show if expsr_othera2=Yes |
| Exposure to Intervention Activities | expsr33 | In the past 6 months, have you participated in individual counseling or psychological support related to HIV? | Yes  No |  |
| Exposure to Intervention Activities | expsr34 | Where did you participate in this individual counseling?  *[Mark all that apply]* | In my neighborhood  At MODEMU  COTRAVED  TRANSSA  IDCP  UVI (Unidad de Vacunas)  COIN  SAI  Other (specify)  Doesn’t remember | Show if expsr33=Yes |
| Exposure to Intervention Activities | expsr35 | In the past 6 months, how many times did you participate in these individual counseling sessions? | __ __ | Show if expsr33=Yes  Max 99 |
| Exposure to Intervention Activities | expsr30 | In the past 6 months, have you participated in a support group with other people living with HIV? | Yes  No |  |
| Exposure to Intervention Activities | expsr31 | Where did you participate in this support group?  *[Mark all that apply]* | In my neighborhood  At MODEMU  COTRAVED  TRANSSA  IDCP  UVI (Unidad de Vacunas)  COIN  SAI  Other (specify)  Doesn’t remember | Show if expsr30=Yes |
| Exposure to Intervention Activities | expsr32 | In the past 6 months, how many times did you participate in these support groups with people living with HIV? |  | Show if expsr30=Yes  Interger  Max: 99 |
| Administrative | Int_end | Time of Completion | [H:M] |  |

End of Survey message:

We have arrived at the end of the survey.

Thank you very much for your time and for sharing your experience with us.

*(Make sure to review the survey before saying goodbye to the participant)*
